# Method using *in vivo* quantitative spectroscopy to guide design and optimization of low-cost, compact clinical imaging devices: emulation and evaluation of multispectral imaging systems

**DOI:** 10.1117/1.JBO.23.4.046002

**Published:** 2018-04-09

**Authors:** Rolf B. Saager, Melissa L. Baldado, Rebecca A. Rowland, Kristen M. Kelly, Anthony J. Durkin

**Affiliations:** a University of California, Beckman Laser Institute and Medical Clinic, Irvine, California, United States; b University of California, Department of Dermatology, Irvine, California, United States; c University of California, Department of Biomedical Engineering, Irvine, California, United States

**Keywords:** tissue optics, multispectral imaging, reflectance spectroscopy

## Abstract

With recent proliferation in compact and/or low-cost clinical multispectral imaging approaches and commercially available components, questions remain whether they adequately capture the requisite spectral content of their applications. We present a method to emulate the spectral range and resolution of a variety of multispectral imagers, based on *in-vivo* data acquired from spatial frequency domain spectroscopy (SFDS). This approach simulates spectral responses over 400 to 1100 nm. Comparing emulated data with full SFDS spectra of *in-vivo* tissue affords the opportunity to evaluate whether the sparse spectral content of these imagers can (1) account for all sources of optical contrast present (completeness) and (2) robustly separate and quantify sources of optical contrast (crosstalk). We validate the approach over a range of tissue-simulating phantoms, comparing the SFDS-based emulated spectra against measurements from an independently characterized multispectral imager. Emulated results match the imager across all phantoms (<3% absorption, <1% reduced scattering). *In-vivo* test cases (burn wounds and photoaging) illustrate how SFDS can be used to evaluate different multispectral imagers. This approach provides an *in-vivo* measurement method to evaluate the performance of multispectral imagers specific to their targeted clinical applications and can assist in the design and optimization of new spectral imaging devices.

## 1 Introduction

Diffuse optical spectroscopy has been demonstrated as a noninvasive technique that can be used to quantify multiple functional and structural properties of tissue. In particular, the wavelength-dependent absorption of light within tissue can be correlated to concentrations of oxy- and deoxyhemoglobin, melanin, lipids, water, among others.^1^^–^^3^ Similarly, the wavelength-dependent elastic scattering of light can be related to subcellular and extracellular components within tissue.^4^

Multispectral imaging is one strategy within diffuse optical spectroscopy that exploits these sources of optical contrast in imaging a spatially resolved paradigm, typically employing planar or structured illumination coupled with a camera. There has been recent increased interest in this approach due to several technological advancements in the size, cost, portability, and/or spectral range of these imaging sensors and sources. Size plays a critical role in translational device development as clinical space is limited and these devices need to be easily accessible in order not to impede clinical flow. Similarly, cost and portability also plays a role in translating biomedical imaging methods into areas of world health and low resource settings. However, with these advances in technology, the emphasis has been on size and cost, leaving several other challenges in instrument design to be considered when developing instrumentation to address unmet clinical needs.

One consequence of adopting an imaging approach, however, is that the number of spectral bands is often limited. Multispectral imagers typically will only offer discrete spectral bands, ranging from 2 to 99 distinct wavelengths. The spectral resolution of these bands may also be quite broad; each band may have 10 to 100 nm bandwidths [full-width at half-maximum (FWHM)].^5^^,^^6^ The spectral resolution of a particular imager may be broader than the spectral features in the extinction coefficient of tissue chromophores, thereby reducing the technique’s ability to differentiate the spectral signatures between distinct tissue chromophores (e.g., oxy- versus deoxyhemoglobin). As a result of this limitation, the accuracy of multispectral imaging becomes heavily dependent on (1) ability to assume accurate knowledge of the chromophores *a priori* that are present in the tissue that will contribute to measured signal (i.e., what we will refer to as “completeness” within the context of this paper) and (2) optimal selection of spectral bands and resolution that can robustly separate and quantify the concentration of each chromophore of interest (i.e., what we will refer to as the minimization of spectral “cross-talk” within the context of this paper).

In contrast to typical multispectral imaging approaches, we have developed a technique called spatial frequency domain spectroscopy (SFDS). This is a quantitative spectroscopic technique that can characterize the optical properties (i.e., absorption and scattering) of tissue from ∼400 to 1100 nm at 1-nm spectral resolution (described in more detail in Sec. 2.1). SFDS can provide far greater spectral detail from tissue than can most multispectral imaging systems, which typically have relatively few, sparsely spaced spectral bands. However, rather than conventional two-dimensional camera-based sensing, SFDS is typically implemented as a point^7^ or line imaging^8^ reflectance spectroscopy geometry. SFDS trades the spatial capabilities (field-of-view and spatial resolution) prevalent in multispectral imagers for enhanced spectral resolution and spectral range. While it may be challenging to fully translate an SFDS imaging system into clinical settings,^8^ the spectral detail of this approach provides a unique platform for the evaluation and potential optimization of wide-field multispectral imagers.

To that end, we have developed a methodology that utilizes the spectral resolution and range of SFDS to evaluate and potentially inform the design of multi-/hyperspectral imaging systems and ensure that these clinical imagers will maintain sufficient spectral content to address the specific needs in their respective targeted biomedical applications.

In this paper, we present a method to simulate the spectral performance of both existing and virtual spatial frequency domain imaging (SFDI) devices, based on measurements obtained using a well-characterized SFDS device. As this approach utilizes *in vivo* measurements, it allows for the analysis of instrument performance for multiple tissue types and structures while still accounting for complex structures and/or biological processes that may not be fully characterized or anticipated *a priori*. Specifically, we present the method to transform our 1-nm resolution spectroscopic measurements (SFDS) into broader spectral responses that may be commonly found in an array of low-cost and/or compact multispectral imaging approaches. This method is validated via its application to a known, previously characterized, multispectral imager. Additionally, we demonstrate the utility of the method within the context of two *in vivo* tissue examples that highlight the challenges associated with *a priori* assumptions of tissue chromophores (e.g., spectral “completeness” and “crosstalk”). While the scope of this paper focuses on SFDI-based multispectral imaging (hence the necessity of SFDS), the underlying method encompasses the case where there is only planar (flood) illumination imaging. Here, simpler point spectroscopy instrumentation may be used instead of SFDS. Implications of extending this approach will be discussed in Sec. 4.

## 2 Methods

### 2.1 Spatial Frequency Domain Spectroscopy

**Fig. 1:**
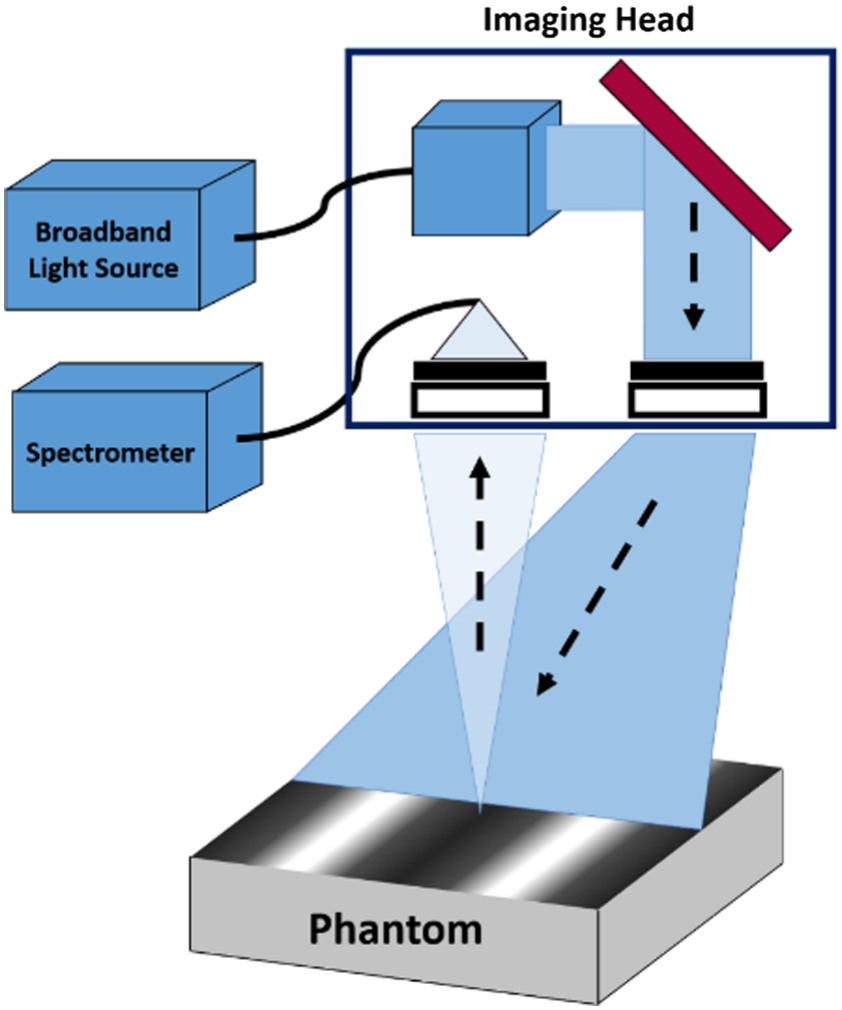
(a) Schematic of SFDS using a broadband light source for illumination.

We have previously reported the details of the SFDS system employed here.^7^^,^^9^^–^^11^ Figure 1 shows the schematic of this SFDS instrument. A 100-W quartz tungsten halogen, QTH, light source (MHF-D100LR, Moritex) was coupled to a digital micromirror device, or DMD, (Alligator XGA DMD module, GFMesstechnik, Berlin Germany) projecting sinusoidal intensity patterns onto an area of 22×17  mm. This instrument can project up to 50 distinct spatial frequency patterns over a range of 0 to $0.5\;{\mathrm{mm}}^{-1}$. Each spatial frequency pattern was projected onto the illumination field three times, each with different phase shifts: 0 deg, 120 deg, and 240 deg, resulting in a total of up to 150 illumination patterns. Typically, only two to seven spatial frequencies are used. The diffuse reflected light from a 1-mm diameter spot size in the center of the illumination field-of-view was collected using a 1-mm core optical fiber and delivered to a spectrometer (Oriel 77480). The spectral response from the imaged sample was recorded as a function of spatial frequency at each phase from 400 to 1100 nm.

Using MATLAB, three phase demodulation of the measured spectra at each spatial frequency is performed in accordance with the procedure that we have previously described.^7^^,^^12^ The demodulated spectra are then calibrated against a diffuse tissue simulating phantom having known optical properties, producing diffuse reflectance spectra as a function of spatial frequency. A scaled white Monte Carlo simulation with discrete absorption weighting is used to model the spatial frequency-dependent reflectance as a function of absorption and reduced scattering coefficients. A nonlinear search function (fminsearch.m) is then used to minimize error between the Monte Carlo model and measured spatial frequency-dependent reflectance at each individual wavelength. This step yields in a unique pair of absorption and scattering values determined at each wavelength independently.^7^^,^^12^

### 2.2 Analysis Method/Generation of Emulated Multispectral Data

In order to simulate the spectral response function of a multispectral imager, the inner product of the SFDS measurements is taken with respect to functions that describe discrete spectral bands of that imager (e.g., LED spectral bandwidths/center wavelengths).

Here, the spectral function, $F_{\lambda i}(\lambda )$, represents the area normalized spectral response/sensitivity for each spectral band (sum to one) used to create the weighted average response for each band, given its spectral shape. These spectral functions can either be directly determined from an existing instrument or can be modeled based on the characteristics/performance of known illumination sources (e.g., LEDs) or on the sensitivity of the detectors (filters, hyperspectral imaging sensors, etc.). It is worth noting that absolute irradiance/detector sensitivity measurements are not necessary, as the instrument response function will be subsequently characterized and compensated for in the calibration stage [Eqs. (4) and (5)].

#### 2.2.1 Generation of emulated measured spectral response from sample/reference

Once the spectral function of each wavelength band has been defined over the entire range of the SFDS measurement (400 to 1100 nm), an emulated measured response, $S_{\mathrm{em}}(\lambda_{I},f_{x},\theta )$, is generated for each spectral band $F_{\lambda i}(\lambda )$ at every spatial frequency ($f_{x}$) and phase (θ) acquired, as shown in Eq. (1) below:

$$S_{\mathrm{em}}(\lambda_{i},f_{x},\theta )=\underset{j}{\sum }S_{\mathrm{SFDS}}[(\lambda_{j},f_{x},\theta )]*F_{\lambda i}(\lambda_{j}).$$ (1)

#### 2.2.2 Demodulation and calibration of emulated SFDI data

In the context of spatial frequency domain measurements, both sample and the reference measurements are demodulated, such that the spatial frequency-dependent reflectance can be determined for both sample and reference:

$$M_{\mathrm{ac}}(\lambda_{i},f_{x})=\;\frac{\sqrt{2}}{3}\sqrt{{\left[S_{\mathrm{em}}(\lambda_{i,},f_{x},\theta_{1})-S_{\mathrm{em}}(\lambda_{i},f_{x},\theta_{2})\right]}^{2}+{\left[S_{\mathrm{em}}(\lambda_{i},f_{x},\theta_{2})-S_{\mathrm{em}}(\lambda_{i},f_{x},\theta_{3})\right]}^{2}+{\left[S_{\mathrm{em}}(\lambda_{i},f_{x},\theta_{3})-S_{\mathrm{em}}(\lambda_{i},f_{x},\theta_{1})\right]}^{2}.}$$ (2)

This demodulated spectral data is then calibrated against the known optical properties of the reference measurement to remove spectral throughput properties of the instrument itself. This stage requires that the optical properties (i.e., absorption and reduced scattering coefficients) of the reference have been previously determined across the 400 to 1100 nm range at 1 nm for the SFDS measurement. In the context of the emulated measurement data, however, the inner product of the known optical properties of the reference is also taken with respect to the spectral function to ensure the reference reflectance model ($R_{\text{model}\_\mathrm{ac}}$) accounts for the spectral response function specific to the multispectral imager:

$$R_{\text{model}\_\mathrm{ac}}{\left(\lambda_{i},f_{x}\right)}_{\text{reference}}=\underset{j}{\sum }R_{\text{model}\_\mathrm{ac}}{\left(\lambda_{j},f_{x}\right)}_{\text{reference}}*F_{\lambda i}(\lambda_{j}),$$

$$\text{Calibrated reflectance}(\lambda_{i,},f_{x})=\frac{M_{ac}{\left(\lambda_{i},f_{x}\right)}_{\text{tissue}}*R_{\text{model}\_\mathrm{ac}}{\left(\lambda_{i},f_{x}\right)}_{\text{reference}}}{M_{\mathrm{ac}}{\left(\lambda_{i},f_{x}\right)}_{\text{reference}}}.$$ (4)

Special case: Calibration of planar (flood) illumination-based reflectance imagers.

Imaging devices that collect only planar reflectance are equivalent to spatial frequency domain approaches, when only the zeroth spatial frequency is considered. In this case, SFDS can emulate the calibrated reflectance of these instruments. For example, if we assume that a 99% Spectralon target (Labsphere, Inc.) is used as reference, then

$$\text{Calibrated Reflectance}(\lambda_{i,})=\frac{M_{\mathrm{ac}}{\left(\lambda_{i},0\right)}_{\text{tissue}}*0.99}{M_{\mathrm{ac}}{\left(\lambda_{i},0\right)}_{\text{reference}}}.$$ (5)

#### 2.2.3 Inverse solver for fitting emulated SFDI data to extract optical properties (mua/musp)

After the emulated reflectance spectra are calibrated, these data are input to an inverse solution model to determine the unique pair of absorption and reduced scattering coefficients that best fit the reflectance data using a model of light transport in tissue. In this study, we have used a scaled white Monte Carlo model to fit for optical properties. We commonly employ this model as it is not dependent on any diffusion approximation and hence provides robust determination in both visible and near infrared spectral regimes.^7^^,^^12^

#### 2.2.4 Fitting emulated absorption spectra to chromophores

Once absorption and reduced scattering coefficients have been calculated, there remains one last stage for which the spectral response function must be utilized. This stage entails the process of determining the concentration of various chromophores by executing a least-squares fit of chromophore extinction coefficients to the wavelength-dependent absorption coefficient. This last stage is particularly critical when visible wavelength bands are considered, as the spectral response function of the imager may be broader than the spectral features of the extinction coefficients for the chromophores present in tissue. This mismatch will result in a reduction in the separability between these chromophores, which will, in turn, result in difficulties related to accurately determining chromophore concentrations. In this stage, the spectral basis set (e.g., extinction coefficient spectra for chromophores, such as melanin, oxy- and deoxyhemoglobin, lipids, water, etc.) is also taken as the inner product with respect to the spectral function of the multispectral imager:

$${\text{Basis spectra}}_{\mathrm{em}}(\lambda_{i},)=\underset{j}{\sum }\text{Basis spectra}[(\lambda_{j})]*F_{\lambda i}(\lambda_{j}).$$ (6)

We have implemented a linear least-squares spectral fitting method to estimate chromophore concentrations in all *in vivo* tissue measurements, as commonly done for bulk (homogeneous) tissue analysis.^7^^,^^10^ In this investigation, the spectral decompositions across all emulated systems are evaluated with respect to the original SFDS absorption spectra.

### 2.3 Characterization of Multispectral Imagers

An SFDI instrument primarily consists of a light source, a digital projector, and a detector. However, there are several approaches for the design and set up of an SFDI instrument and its components to adjust spectral range, spectral resolution, acquisition speed, and cost. In the past, broadband light sources and filters (i.e., liquid crystal tunable filters) have been used to project light at wavelengths in the visible and/or NIR regime.^12^^–^^16^ More recently, some SFDI devices employ light-emitting diodes (LEDs), where each LED has distinct center wavelength and bandwidth characteristics, to accomplish the same goal.^17^^–^^20^

In the context of this study, we investigate the performance of three distinct multispectral SFDI approaches, using the methodology that we have presented. One of these instruments uses a series of LED sources and a monochrome camera to detect the light remitted from tissue. This instrument, OxImager RS (Modulated Imaging, Inc), is used as an example with which to test the accuracy of the spectral emulation method (Sec. 2.3). The other two instruments utilize spectral filtering on the detection side of the instrument. Here, a generic color camera using a Bayer filter (three-color imager) and a specialized Fabry–Perot filter mosaic-based imager (XiSpec, Ximea/IMEC hyperspectral snapshot imager) are considered.

#### 2.3.1 OxImager RS

The OxImager RS was the first device investigated using the method described here. Similar to the SFDS system previously described in Sec. 2.1, the OxImager RS images the spectral response obtained from turbid media across both visible and NIR. The OxImager RS, however, has several key distinctions as to how it is implemented. While the SFDS system utilizes broadband illumination as its source and a spectrometer to detect the spectral response at 1-nm resolution, the OxImager RS serially illuminates the sample through a sequence of eight discrete LEDs. Light remitted from the sample region of interest is detected by a monochrome CCD. The spectral bandwidth of each LED can range from ∼20 to 30 nm. While the spectral properties may be reduced relative to SFDS, the OxImager RS offers a 20×15  cm field-of-view that allows for the quantitative mapping of spatially resolved chromophore concentration of large tissue regions of interest.

To confirm the source illumination spectral properties used in the OxImager RS, the LED spectral bands were measured. In order to do this, the LED output from the OxImager was coupled into a fiber, which was coupled to the entrance slit of a spectrometer (FLAME-S-VIS-NIR, Ocean Optics). The measured response from the device was imported into MATLAB and interpolated to match the spectral resolution of the SFDS system and normalized (such that the sum of the spectral response of each LED equals unity). These spectral functions were then incorporated into our analysis code to simulate the spectral response functions of the OxImager.

#### 2.3.2 Three-color imager

Our emulation approach was also applied to a three-color imager in order to evaluate its performance if used in an SFDI system. For this example, we measured the spectral sensitivity of a webcam (Live! Cam Notebook Ultra, Creative, Inc.) having a Bayer filter with red (R), blue (B), and green (G) channels. The red, green, and blue channels have different sensitivities to light. Our test set up consisted of a broadband light source (HL-2000, Ocean Optics) coupled to an optical fiber (200  μm, 0.22 NA, Thorlabs), a holographic transmission diffraction grating (#54-510, 500  lines/mm, Edmund Optics), and the webcam. Here, the webcam images the distal end of the 200-μm fiber. By fixing the transmission grating in front of the webcam, both the zeroth and first order diffraction of broadband light can be captured by the webcam. From the first-order diffraction of light, the spectral sensitivity of each color channel can be determined. Additionally, lasers at 633 and 532 nm were also used in this setup to verify the spectral calibration of this simple transmission grating spectrometer.

#### 2.3.3 XiSpec hyperspectral snapshot imager

For this example, we emulated the performance of a hyperspectral snapshot imager that uses a mosaic hyperspectral sensor (XiSpec, IMEC/Ximea). Similar to the Bayer filter used in the previously mentioned webcam example, the mosaic hyperspectral sensor utilizes an array of filters having different spectral sensitivities for each wavelength channel. However, unlike the Bayer filter used in RGB imagers, this hyperspectral imager utilizes a mosaic of Fabry–Perot cavities to generate wavelength-selective spectral channels. This specific sensor has 16 narrow bands in the visible range from 430 to 630 nm with bandwidths less than 15 nm. As these are custom designed imagers, each sensor will have a unique spectral performance characteristic.^21^ Using the sensitivity functions provided by the manufacturer for a specific sensor, we were able to emulate the behavior of a snapshot hyperspectral imager using our code.

### 2.4 Accuracy of Emulation Code

To test the accuracy of the multispectral emulation code, we implement the emulated spectra method utilizing the spectral functions of the OxImager RS and then compare these emulated data responses to those measured directly using the device itself.

#### 2.4.1 Homogeneous phantoms

Three homogenous tissue simulating phantoms with different optical properties were measured using both SFDS and SFDI systems and compared (optical properties shown in the Sec. 3, Fig. 4). Three dyes were used as absorbing agents so that not only the magnitude of absorption can vary between these phantoms, but each would also exhibit a distinct spectral shape. One phantom uses nigrosin as the absorbing agent and titanium oxide as the scattering agent. This phantom has absorption values ranging from 0.23 to $0.01\;{\mathrm{mm}}^{-1}$, with its maximum value at ∼600  nm and its minimum at ∼850  nm. A phantom that uses naphthol green as the absorbing agent and titanium oxide as the scattering agent was used to show our method could properly quantify relatively higher absorption properties of naphthol green in the near-infrared regime ($∼0.03\;{\mathrm{mm}}^{-1}$) over that in the visible ($∼0.02\;{\mathrm{mm}}^{-1}$). This phantom also contained approximately half the titanium oxide, resulting in half the scattering than the other phantoms. Last, a phantom that uses India ink as the absorber and titanium oxide as the scattering agent was used to demonstrate our method’s ability to measure weak ($0.02\;{\mathrm{mm}}^{-1}$) homogenous absorption across both visible and near-infrared regimes while maintaining higher reduced scattering optical properties.

All three phantoms were measured using both the SFDI (OxImager RS) and the SFDS systems. Both instruments collected data using five spatial frequencies (0, 0.05, 0.1, 0.15, and $0.2\;{\mathrm{mm}}^{-1}$) and three phases (0 deg, 120 deg, and 240 deg). The SFDI instrument imaged the phantoms at seven wavelengths centered at 471, 529, 591, 659, 691, 731, and 851 nm, whereas the SFDS system collected the broadband spectral response from 400 to 1100 nm at 1-nm spectral resolution. Using the multispectral emulation method that we have described, the raw SFDS measurements were convolved with the measured LED functions of the SFDI instrument.

### 2.5 In Vivo Tissue Case Studies (Homogeneous Interpretation)

After completing the phantom oriented studies, we illustrate the technique within the context of two small sets of *in vivo* data: (1) wound progression due to thermal injury in a preclinical model and (2) imaging of changes in human skin due to sun exposure. Most multispectral imagers have limited numbers of spectral bands and resolution; highlighted here, the OxImager RS uses 8 spectral bands, the XiSpec hyperspectral snapshot imager has 16 bands while the color camera has 3. Hence, it is important to verify that these devices collect sufficient spectral information needed to separate and quantify the physiologically relevant chromophores of interest. Particular chromophores such as oxy- and deoxyhemoglobin can be used as biomarkers for tissue viability in some specific applications (e.g., burn wounds). It is also important to ensure that these devices account for additional, uncorrelated sources of optical contrast that may confound the diagnostically relevant information (e.g., accumulated sun exposure: photoaging).

Here, spectral analysis of a preclinical burn wound progression model has been used to illustrate whether a multispectral imager has sufficient number of spectral bands and resolution to robustly isolate and quantify the chromophores present in tissue (i.e., assessment of spectral cross-talk), and a preliminary dataset examining photoaging of skin has been used to illustrate errors that may occur when additional and/or unanticipated chromophores are present in the *in vivo* measurement.

#### 2.5.1 Monitoring burn wound progression in a preclinical model

*In vivo* data were acquired from a rat model under an approved UC Irvine Institutional Animal Care and Use Committee protocol (IACUC #1999-2064). A 313-g “brass comb” was used to induce homogeneous burn wounds on male CD hairless rats weighting between 300 and 450 g.^14^^,^^22^ The animal was imaged at baseline, before application of the burn, 80 min after the burn, and 72 h after the burn using the SFDS system to evaluate the hemodynamic changes in the tissue over time.

All SFDS measurements were performed at seven spatial frequencies evenly spaced from 0 to $0.36\;{\mathrm{mm}}^{-1}$. The data were processed using both the standard SFDS method and the emulation approach to evaluate errors associated with unaccounted for chromophores. For the purpose of this case study on multispectral imager performance, the measured and emulated spectral data are compared on a 20-s burn, corresponding to a histologically verified superficial partial thickness burn in the context of a murine skin.^14^^,^^22^

#### 2.5.2 Photoaging of skin; the presence of uncorrelated biological variance in skin composition

In this study, we examine how multispectral imaging devices may perform in an examination of the accumulated effects of sun exposure on skin (i.e., photoaging). The process of photoaging will not only effect the chromophore concentrations in skin (e.g., melanin, oxy- deoxy, etc.) but also effect structural aspects as well (e.g., collagen).^23^ Given that there is a wide variance in optical properties in normal skin tissue (range in pigmentation, blood volume, oxygenation, etc.), accurate determination of these properties is critical in order to isolate and quantify the secondary effects that arise from photoaging. These effects may include degradation of connective tissue integrity, changes in pigmentation, inflammation, and fibrosis.^24^ In order to account for these changes, some of which are subtle, all potential (independent/tertiary) sources of absorption must also be considered.

Carotenoids, which are organic pigments that are produced by plants, reflect aspects of dietary plant consumption.^25^ Carotenoids present an interesting challenge to quantitative imaging of skin in the visible spectrum as they typically are, in the context of healthy subjects, a consequence of dietary intake and not necessarily related to the targeted progression of tissue pathology. Carotenoids are lipophilic and typically reside within tissue structures that are millimeters deep (i.e., subcutaneous fat) and therefore would not be detected by visible light that only interrogates hundreds of microns into tissue. However, these compounds can also be found in dermal appendages, such as sebaceous glands, hair follicles, and blood serum.^25^

In most skin imaging publications, however, accounting for this absorption contribution is not considered, nor discussed, unless it is exclusively the targeted objective of the study.^26^^–^^28^ This is true for our prior publications on skin imaging as well.^9^^,^^10^ For this reason, we consider how the absorption features from carotenoids (450 to 490 nm) may impact the interpretation of *in vivo* skin under conditions of sun exposure and nonsun exposure.

Utilizing this approach to generate emulated multispectral data from actual SFDS measurements, we evaluate the potential accuracy of the multispectral imagers to replicate the chromophore concentration results obtained by SFDS: melanin, carotenoids, oxy- and deoxyhemoglobin, and the reduced scattering spectra (characterized as the amplitude and slope of a power-law, $A\cdot \lambda^{-b}$).^4^ We consider preliminary SFDS measurements of the dorsal forearm (sun exposed) versus the inner upper arm (nonsun exposed) of six subjects ranging in age from 20 to 50 years old (N=12). These subjects were limited to Caucasian skin types within the scope of this initial study, types I-II on the Fitzpatrick scale.^29^ These data was acquired under an IRB approved protocol: #2008-6307. Seven spatial frequencies, ranging from 0 to $0.36\;{\mathrm{mm}}^{-1}$, were used to generate the absorption and reduced scattering spectra.

## 3 Results

### 3.1 Spectral Functions of Emulated Multispectral Imagers

**Fig. 2:**
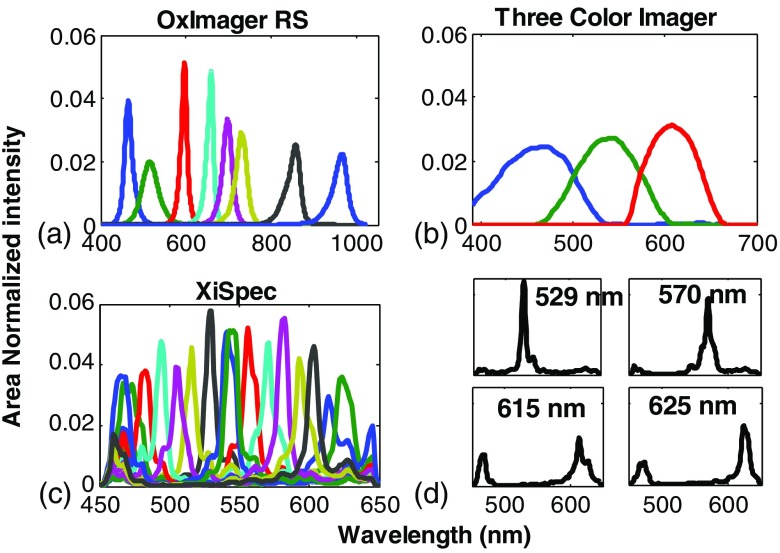
Spectral response functions for the emulated imagers: (a) the spectral bandwidth of the eight LEDs used in the OxImager, (b) the spectral sensitivity of the Bayer filter from the color imager, and (c) the spectral sensitivity of the 16 channels of the XiSpec hyperspectral snapshot imager. Panel (d) calls out four individual channels from the XiSpec imager, illustrating the best (top figures) and worst (bottom) cases of spectral selectivity from this imager.

Figure 2 shows the spectral functions that used to describe the three multispectral imaging approaches: the OxImager RS, a three-color imager, and a XiSpec hyperspectral snapshot imager. The OxImager’s spectral resolution is primarily defined by the bandwidth of the series of LEDs employed to sequentially illuminate tissue. Here, the FWHM of these LEDs range from 44 nm (529 nm) to 15 nm (591 nm). Because the spectral function is presented under area normalization, the amplitude of each channel is also an indication of its resolution (i.e., the higher the peak, the narrower the resolution). The color camera (RGB imager) displays three spectral bands in the visible regime only, where the center wavelengths are 470, 540, and 610 nm and the FWHMs range from 95 to 67 nm.

The XiSpec, however, has a significantly more complex spectral function [Fig. 2(d), top panels]. While the majority of spectral channels exhibit significantly higher spectral resolution relative to the other imagers considered in this study, there remains an underlying issue with the out-of-band rejection in each of these channels. Although small, there remains some contribution (out of band transmission) from nearly all wavelengths across the visible spectral regime [Fig. 2(d), top panels]. Integrated across the entire visible spectral region (450 to 700 nm), this out-of-band contribution can represent ∼35% of the total signal measured under the best case in this particular sensor (529-nm channel). Some other channels, however, exhibit a multiband spectral sensitivity [Fig. 2(d), bottom panels]. Here, the channels will detect spectral contributions from two distinct spectral regions.

### 3.2 Method Validation

#### 3.2.1 Homogenous phantoms (reflectance)

**Fig. 3:**
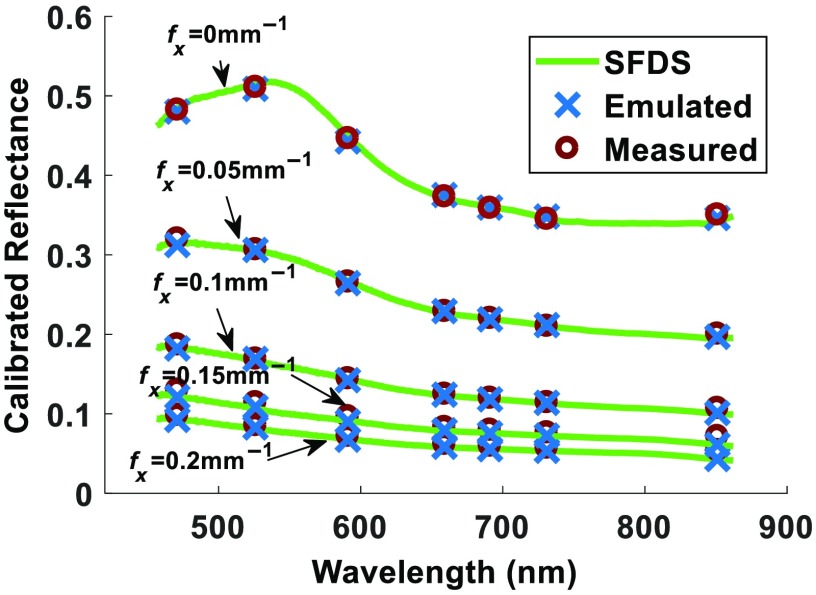
Calibrated reflectance of a homogeneous tissue simulating phantom that uses naphthol green as the absorbing agent and titanium oxide as the scattering agent processed using SFDS, SFDI, and the emulation method.

The normalized intensities of the measured LEDs of the OxImager are shown in Fig. 2(a). Figure 3 shows the calibrated reflectance of a naphthol green phantom measured and processed using three methods. The green lines show the resulting reflectance spectra at each spatial frequency using the standard SFDS instrumentation and processing methods with 1-nm resolution. The red circles illustrate the calibrated reflectance at each wavelength as a function of spatial frequency using the OxImager RS, and the blue points are the results from the multispectral emulation method using the measured LED functions. Differences in calibrated reflectance between the emulated and measured OxImager data were within 1% agreement across all spatial frequency-dependent reflectance spectra.

#### 3.2.2 Homogeneous phantoms (absorption and scattering)

**Fig. 4:**
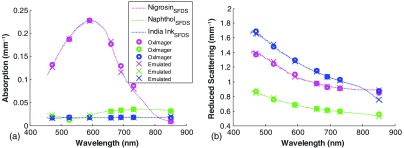
Measured optical properties of three homogeneous tissue simulating phantoms using SFDS, SFDI, and emulated multispectral data methods. (a) Absorption coefficient and (b) reduced scattering coefficient. Dotted lines are the results after measuring and processing with standard SFDS methods. The results from using SFDI are depicted by circles and the emulated multispectral data points are shown as an “x.”

Figure 4 shows the measured optical properties of three tissue simulating phantoms using the SFDS, OxImager, and emulated methods. Each phantom uses a different dye as its absorbing agent (Nigrosin, Naphthol Green B, and India Ink), as well as different concentrations of titanium oxide (${\mathrm{TiO}}_{2}$) as the scattering agent. These phantoms were selected for this study as each has distinct spectral absorption features, and the combination of absorption and scattering properties span a range of tissue optical properties typically encountered. The dotted lines represent the results using the standard SFDS process. The circles are the results from measuring the phantoms with the OxImager. The resulting optical properties using our multispectral emulation method are denoted with the symbol “x.” Differences in the calculated absorption coefficient over all phantoms between the OxImager RS and emulation processing method are within 3%. Differences in the calculated reduced scattering coefficient between the OxImager data and the emulated spectra are within 1% across all phantoms. The results for each phantom are presented in Table 1.

**Table 1:** Error in calculated optical properties between OxImager RS measurements and the SFDS-based emulation of the OxImager.

Phantom	Absorption coefficient (%)	Reduced scattering coefficient (%)
India Ink	±1.4	±0.34
Napthol	±2.8	±0.53
Nigrosin	±1.9	±0.24

### 3.3 Reduced/Modified Chromophore Basis Sets Due to Imager Spectral Functions

**Fig. 5:**
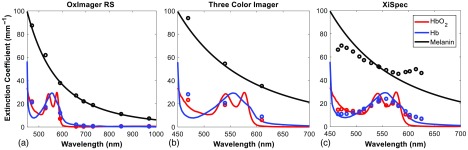
Reduced spectral response from selected chromophores due to the limited spectral resolution of the imaging devices that have been chosen for emulation. Each panel shows the extinction coefficients of melanin, oxy- and deoxyhemoglobin at 1-nm spectral resolution (solid line) and after their inner product is taken with respect to the emulated imager (open circles): (a) OxImager, (b) color imager, and (c) XiSpec.

Figure 5 illustrates the change in the spectral absorption values of chromophores when the inner product (Sec. 2.1) with the spectral function of the three imagers is considered, with respect to the 1-nm resolved reference spectra of these chromophores (solid lines). For clarity, only reference spectra from melanin, oxy- and deoxyhemoglobin have been included in this figure.

In the case of both OxImager and color imager, there is a noticeable reduction in separation between oxy- and deoxyhemoglobin reference values (solid lines) in the visible regime. This is a consequence of the spectral function from these imagers being broader than the spectral features (peaks) of chromophores in this wavelength regime. The OxImager has its broadest spectral resolution at 529 nm, which happens to occur near the alpha absorption band of oxyhemoglobin [Fig. 5(a)]. The FWHM of the LED is 44 nm, yet the alpha band feature is ∼25  nm wide. Similarly, the 540-nm spectral band of the three-color imager is coincident with the alpha absorption band, however, it is 74-nm wide (FWHM). As a result, the difference between the extinction coefficients of oxy and deoxyhemoglobin at this spectral band is reduced by over a factor of 2 [Fig. 5(b)]. The XiSpec also exhibits a departure from the pure reference chromophore extinction spectra [Fig. 5(c)], however, this redefinition of chromophore reference spectra is a consequence of the reduced out-of-band rejection (over the central wavelengths, 470 to 600 nm) and the dual wavelength sensitivity present in the shortest and longest wavelength regimes (450 to 470 nm, 600 to 650 nm). The consequence of this spectral selectivity is most apparent in the reduced melanin spectrum, where clear departures from the power-law like extinction coefficient are evident in the short and long wavelength segments (as well as a reduction in spectral slope due to the reduced out-of-band rejection).

### 3.4 In Vivo Tissue Examples

#### 3.4.1 Preclinical burn wound monitoring

**Fig. 6:**
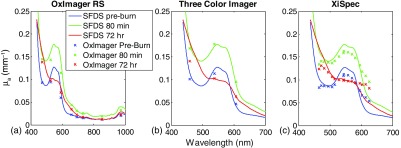
Burn wound progression over a 72-h period, as calculated from the spectral functions each of the emulated imagers. Here, solid lines in all panels represent the absorption spectra measured *in-vivo* by SFDS, “x” indicates the emulated spectral response where it is measured by the (a) OxImager, (b) color imager, and (c) XiSpec.

Figure 6 shows the measured absorption data from three distinct time points in the burn wound protocol: (1) baseline (preburn), (2) 80-min postcreation of a histologically verified deep partial thickness burn, and (3) 72-h postburn injury. Each panel of Figs. 6(a)–6(c) illustrates how this spectral response would be captured by the three multispectral imagers emulated in this study. From the SFDS absorption spectra, contributions from oxy and deoxyhemoglobin are readily apparent (contributions from melanin are present, but minimal, since this strain of hairless rats does not exhibit strong pigmentation). After 72 h, however, the wound has subsequently progressed, resulting in changes to the absorption spectrum that suggests the evolution of additional chromophores (as can be seen in the red spectrum across all figure panels). This is most noticeable in the increased absorption feature within the 600 to 700-nm spectral range.

In order to more completely analyze this, we introduced chromophore extinction coefficient line shapes into the fitting that are consistent with these hemoglobin breakdown products. In particular, we have included methemoglobin^30^ in the fitting across the entire time series and note that this is even present (in a smaller concentration) within 80 min of the thermal injury.

As multispectral imagers generally collect sparse spectral information and are dependent on *a priori* assumptions regarding which chromophores are present, the generation of these breakdown products may go unnoticed, resulting in misestimation of hemoglobin species and/or tissue status. Table 2 provides tissue oxygenation and methemoglobin values that result from the spectral sensitivities of each imager, when it is assumed that oxy-, deoxy-, and methemoglobin are present in the tissue along with melanin (OxImager and XiSpec) and water (OxImager). The SFDS values are also presented as reference. Here, both OxImager RS and the XiSpec snapshot imager are able to generate trends that match the relative changes in both tissue oxygenation and hemoglobin breakdown products. Additionally, the actual values from these devices also follow closely to SFDS (∼10% for ${\mathrm{StO}}_{2}$ and ∼20% for MetHb). In the case of the three-color imager, however, tissue oxygenation is consistently underestimated (∼20% to 12%), due to the fact that with only three spectral channels, we have elected to fit absorption to the three species of hemoglobin and not consider the contribution from melanin. Assuming that the, although small, contribution from melanin would not affect the determination of the three dominant chromophores present bears some consequences, as illustrated by this case study.

**Table 2:** Tissue oxygenation and relative MetHb percentage assess by all spectral imagers. Here, MetHb is reported as a percentage relative to all hemoglobin species: $\mathrm{MetHb}/({\mathrm{HbO}}_{2}+\mathrm{Hb}+\mathrm{MetHb})$, whereas ${\mathrm{StO}}_{2}$: ${\mathrm{HbO}}_{2}/({\mathrm{HbO}}_{2}+\mathrm{Hb})$.

	Preburn		80-min postburn		72-h postburn	
	${\mathrm{StO}}_{2}$ (%)	MetHb (%)	${\mathrm{StO}}_{2}$ (%)	MetHb (%)	${\mathrm{StO}}_{2}$ (%)	MetHb (%)
SFDS	74	2.7	89	12	95	25
OxImager RS	75	3.2	87	11	92	23
Three-color imager	65	3.4	68	12	79	16
XiSpec	78	2.5	81	14	90	20

#### 3.4.2 Photoaging of skin; influence of carotenoids

**Fig. 7:**
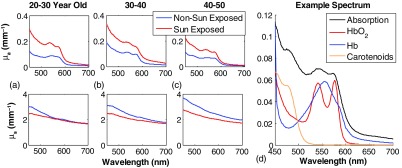
Age differentiated optical properties from sun exposed and nonsun exposed skin tissue. Panels (a), (b), and (c) show the mean absorption (top) and reduced scattering (bottom) as a function of age group. The spectra indicated in blue represent nonsun exposed tissue (volar upper arm) and the spectra indicated in red represent sun exposed (dorsal forearm). Panel (d) shows the spectral components of an example measured absorption spectrum (black); melanin is not shown. While only data from 450 to 700 nm are shown here, the measured dataset spans 435 to 1050 nm.

In this case study, we contrast the results from the three emulated multispectral imagers toward the quantification of optical properties related to normal skin, sun exposed (dorsal forearm) versus nonsun exposed [inner (volar) upper arm]. Figure 7 shows the mean absorption and reduced scattering spectra from sun and nonsun exposed skin. Here, the results are clustered as a function of three age groups: 20 to 30, 30 to 40, and 40 to 50 years old, (1 male and 1 female per group, Caucasian: skin type I-II). Figure 7(d) shows the absorption spectrum from one of these subjects (volar, nonsun exposed upper arm), where there is a distinct spectral feature that appears between the Soret (420 nm) and alpha/beta (580 nm) bands expected from hemoglobin. This additional spectral feature matches that of published spectra from carotenoids.^31^

As these were all healthy subjects, the presence of carotenoids in the absorption data was presumed to be a function of individual dietary habits and not correlated with accumulated sun exposure (photoaging) or age, nor related to any functional or metabolic disorder. There was a consistent differentiation between the scattering parameters (amplitude, A, and slope, b,) between sun exposed and nonsun exposed tissues that suggests agreement with published research that describes accumulated effects of sun exposure on skin.^23^^,^^32^

Table 3 depicts the relative error of each emulated imaging system with respect to the tissue optical parameters extracted from the SFDS measurements across all six subjects and tissue locations acquired (n=12). Here, both the OxImager and the XiSpec are able to replicate results from the full spectral fitting of the absorption data (e.g., melanin, ${\mathrm{StO}}_{2}$, carotenoids) within 20%, whereas the three-channel color imager demonstrated larger bias and variance as it was attempting to fit four chromophores (i.e., melanin, carotenoids, oxy- and deoxyhemoglobin) with only three spectral channels of data. However, if the spectral features from carotenoids are omitted from the spectral analysis of this dataset (parenthetical values in Table 3), then all imagers would overestimate melanin by ∼40% and ${\mathrm{StO}}_{2}$ would be biased toward higher oxygenation levels.

**Table 3:** Errors in estimation of tissue properties relative to the SFDS measurement data. Parenthetical values: errors in melanin and ${\mathrm{StO}}_{2}$ estimation were carotenoids not included in the decomposition of the absorption spectra.

Relative error	Melanin		${\mathrm{StO}}_{2}$		Carotenoids		Scatter slope (b)	
	Variance %	Bias %	Variance %	Bias %	Variance %	Bias %	Variance %	Bias %
OxImager RS	±13(21)	+10(+37)	±11(18)	+6(+13)	±5	+2	±3.2	−3.8
Color imager	±31(45)	+9(+41)	±28(31)	−20(−15)	±23	−47	±1.7	−0.3
XiSpec	±17(25)	−15(+35)	±11(19)	−5(+15)	±14	−5	±3.6	−45

Given the simple power law model for fitting the reduced scattering spectra, both the OxImager and the three-color imager rendered reduced scattering coefficients within 4% and 2% agreement of those that were determined using SFDS data, respectively. While the XiSpec was able to demonstrate a small variance (3.6%) in scattering relative to the SFDS data, there remained considerable bias (−45%) in the parameter fitting of the scattering due to the spectral mixing of the short (450 to 500 nm) and long (580 to 650 nm) wavelength channels.

## 4 Discussion

In this paper, we have presented and evaluated a method for the characterization of multispectral imaging devices in the context of determining whether the spectral content/resolution is sufficient to address the complexity specific to the target biomedical application. This method is designed to enable emulation of reflectance multispectral imaging devices, in general, within the visible and near-infrared regimes. To first verify the accuracy of this approach, the method was used to emulate an existing multispectral device over a range of independently characterized tissue simulating phantoms having various optical properties. The emulated results were then directly compared to the actual measurements collected using the instrument that is subject to the analysis from the same tissue simulating phantoms (Fig. 3: calibrated reflectance and Fig. 4: absorption and reduced scattering). Given errors that may arise from the LED characterization via the method described in Sec. 2.3.1, as well as relative measurement noise/error that may arise between the two SFDI/S devices,^33^ these results remain convincing in that they are able to emulate results within 1% of the spatial frequency-dependent reflectance and within <3% of the extracted absorption and <1% reduced scattering spectral coefficients, spanning from 450 to 970 nm. Here, we have demonstrated that this approach is accurate over an anticipated range of spectral properties for *in vivo* tissues, such as skin.^7^^,^^9^^–^^11^

Quantitative methods for optical spectrographic measurements of *in-vivo* tissue, such as SFDS, are important toward advancing our understanding of the nature of the optical properties present in tissue. While these techniques may be limited with respect to spatial information content, they do provide spectral detail that exceeds that of the sources of optical contrast present in tissue (e.g., tissue chromophores and scattering). Used as an investigational tool, SFDS can characterize both the chemical and structural composition of tissue via the interpretation of the measured absorption (chromophore) and reduced scattering spectra. While multispectral imagers require *a priori* assumptions of chromophores (and spectral shape) in order to interpret the absorption spectrum it measures, SFDS absorption spectra can be used to test whether these assumptions (1) account for all chromophores present in the measurement and (2) whether the functional form (spectral shape) is sufficient to describe each of these components.

Recently, the design and validation of a clinical imaging SFDS system have been published.^8^ Here, it was demonstrated that broadband, wide-field structured illumination could be achieved for under $1000 through the careful selection of a quartz tungsten halogen source and a modified picoprojector. Adding a simple fiber coupled spectrometer as a detector, it is possible to develop an SFDS system for ∼$5  k−10  k. These recent developments make SFDS measurements both approachable and reasonable as a preliminary investigative tool to characterize *in vivo* tissue.

Should the ultimate multispectral imaging instrument only utilize planar illumination (without the addition of spatial frequency dependent reflectance), a fully enabled SFDS instrument may not be required. A noncontact, point spectroscopy measurement geometry may be sufficient. Essentially planar illumination (flood illumination) is the same as SFDI, where the spatial frequency is set to 0. In this simplified case, the same overlap integrals and emulation method would be employed, but the calibration of these data would use Eq. (5) and the subsequent analysis would be based on emulated reflectance, rather than emulated absorption and reduced scattering spectral data.

The two *in vivo* cases presented here (Sec. 3.4) were specifically selected to illustrate the relative performance of SFDS in terms of quantifying chromophore concentration. They also help to illustrate how SFDS can characterize the chromophores present and then evaluate the accuracy of multispectral imagers to match the characterization of these chromophores and tissue scattering properties. The objective of this investigation was not to determine whether one of the emulated multispectral imaging approaches is superior to the others. On the contrary, we believe that each imager holds distinct advantages and disadvantages. Only through a careful understanding of the spectral sensitivity of the imager in the context of the targeted application (e.g., sensitivity to relevant biomarkers in the presence of other sources of spectral variance and tissue structure), we would promote one approach over another.

In the context of burn wounds (Fig. 6), the key chromophores that characterize the functional response to thermal insult discussed here are oxy- and deoxyhemoglobin and the generation of hemoglobin breakdown products that become detectable within an hour of injury. The contribution from melanin was small in this preclinical animal model (∼1%); however, including this chromophore in the fitting of the respective absorption spectra allowed for the consistent estimation of all three hemoglobin species. This is evident in the results summarized by Table 2, where both the OxImager and XiSpec snapshot imager provided consistent estimations of oxy-, deoxy- and methemoglobin relative to SFDS. Melanin was not included in the spectral fitting of the three-color imager. This most notably resulted in an overestimation of deoxyhemoglobin and hence an underestimation of tissue oxygenation. The three-color imager did track a similar trend in relative tissue oxygenation and methemoglobin over the time course of this wound.

Carotenoids present an interesting challenge to quantitative imaging of skin in the visible spectrum as they typically are, in the context of healthy subjects, a consequence of dietary intake and not necessarily related to the targeted progression of tissue pathology. Figure 7(d) illustrates that absorption features that correlate with carotenoid absorption can be measured by optical methods in the visible spectral regime. Table 3 also indicates that if carotenoids are not included in the spectral analysis of visible regime spectral imaging, a large (∼40%) overestimation of melanin can occur. Tissue oxygenation can also be overestimated as the presence of carotenoids may falsely be attributed to oxyhemoglobin rather than deoxyhemoglobin. If these sources of optical contrast are not more readily discussed, there is a chance that they will never be considered in the *a priori* assumptions that used to model tissue in the context of multispectral imagers.

The primary motivation behind including a three-color imager in this investigation is the fact that it is the most prevalent, low-cost multispectral imager available. It is ubiquitous, present in nearly every phone, tablet, and laptop produced today. This has vaulted this type multispectral imaging approach to become the basis for the majority of low-cost solutions for clinical challenges in under-served regions. As illustrated in the two case studies presented here, this class of imager is, however, limited to address the full diversity of sources of optical contrast in tissue; as the spectral resolution of the imager is significantly broader than the spectral absorption features of tissue chromophores in the visible [Figs. 2(b) versus Fig. 5(b)], making it far more important to first demonstrate sufficiency first (e.g., minimize or address confounding sources of optical contrast and isolate sources specific toward the target application).

As was the case with the application of quantitative imaging of photoaging of skin, the three-color imager did not perform well with respect to interpreting its absorption data; however, it did remain quite accurate in terms of describing tissue scattering. From the preliminary dataset presented here, tissue scattering [the scatter slope (b), in particular] was the most highly correlated, subject-specific difference between sun exposed and nonsun exposed skin as a function of accumulated sun exposure. While chromophore concentrations (e.g., melanin and hemoglobin species) can still play roles in characterizing photoaging of skin, they also vary due to genetic and environmental factors and require full accounting of other chromophores present in tissue (e.g., carotenoids) before their contribution could be explained. Scattering spectra, on the other hand, appear to be insensitive to these other contributing factors within the context of this preliminary dataset. While larger studies are required to evaluate the diagnostic potential, reduced scattering spectra are invariant to the presence of carotenoids and, as they are characterized by a simple and smooth power law,^4^ they do not require high spectral resolution and are easily described at three wavelengths. Thus, while the ability to characterize chromophores is limited, the three-color imager may be useful as a low-cost imaging tool to monitor the structural changes in skin resulting from sun exposure.

It is worth noting that several groups have been developing devices that address concerns regarding spectral differentiation of chromophores by modifying the spectral selectivity of these three color devices to increase spectral resolution and/or increase the number of spectral channels under this three-color detection platform.^34^^,^^35^ The key to the need and development of these modifications is an understanding of not just biomolecular changes that relate to the targeted disease or condition but also the concentration and dynamics of unrelated biomolecules as well. The *in-vivo* data-driven approach proposed here aims to provide not only just a basis to identify these biomolecules and characterize their dynamics for this modified three-color imager but also to provide an a priori means to evaluate instrument performance before deploying clinically. There remain several relatively unexplored opportunities that can exploit the low cost nature of these simple imaging platforms that remains beneficial (informative) toward advancing our understanding of tissue properties. These low-cost approaches present practical opportunities to facilitate dissemination of translational devices into multicenter research studies and in low resources settings. This avenue has the potential to create opportunities to collect large-scale clinical studies, based on carefully designed optical parameters.

While the method proposed in this investigation requires SFDS measurements as a basis for the evaluation of the performance of multispectral imaging approaches to address current clinical needs and translational challenges, the ultimate goal is to translate this approach to a purely virtual method. One requirement of this translation is a comprehensive model for tissue that not only encompasses the biological processes and parameters relevant to the target application, but also considerations for the independent factors that may also impact the optical measurement (e.g., variance in skin pigmentation, diet, etc.). While this may seem daunting, given the complexity and diversity of tissue physiology, the continued efforts of several groups^36^^–^^42^ continue to advance and refine our understanding of tissue biology and thereby inform more complete models of tissue structures, constituents, and variance. Additionally, others have provided tools that assist in modeling light–tissue interaction at a computation level.^43^

## 5 Conclusion

We have developed a method to simulate the performance of multispectral imagers based on SFDS. As this method is informed from ∼1  nm spectrally resolved *in vivo* measurements, it affords the opportunity to characterize aspects of the performance of multispectral imaging systems in the context of their target application. In addition to providing an opportunity to identify the prevalent physiological parameters related to the target application, SFDS can also characterize the potential sources of biological variance that may also contribute to multispectral imaging of tissue. This approach, demonstrated here, provides a means for the informed development of viable translational devices into clinical practice.
